# Longitudinal age-and cohort trends in body mass index in Sweden – a 24-year follow-up study

**DOI:** 10.1186/1471-2458-13-893

**Published:** 2013-09-27

**Authors:** Ozge Karadag Caman, Susanna Calling, Patrik Midlöv, Jan Sundquist, Kristina Sundquist, Sven-Erik Johansson

**Affiliations:** 1 Department of Public Health, Faculty of Medicine, Hacettepe University, Ankara, Turkey; 2 Department of Clinical Sciences, Center for Primary Health Care Research, Lund University, Malmö, Sweden; 3 Stanford Prevention Research Center, Stanford University, Palo Alto, California, USA; 4 Center for Primary Health Care Research, Lund University, Clinical Research Centre (CRC) Skåne University Hospital, Jan Waldenströms gata 35, 205 02 Malmö, Sweden

**Keywords:** Age, Birth cohort, Body mass index, Longitudinal data, Mixed models

## Abstract

**Background:**

The aim of this longitudinal study was to analyze whether mean Body Mass Index (BMI), assessed at four occasions, changed within different age groups and birth cohorts over time, i.e., between 1980/81 and 2004/05, after adjustment for possible confounders.

**Methods:**

A sample of 2728 men and 2770 women aged 16–71 years at study start were randomly drawn from the Swedish Total Population Register and followed from 1980/81 to 2004/05. The same sample was assessed on four occasions during the 24-year study period (i.e., every eighth year). The outcome variable, BMI, was based on self-reported height and weight. A mixed model, with random intercept and random slope, was used to estimate annual changes in BMI within the different age groups and birth cohorts.

**Results:**

Mean BMI increased from 24.1 to 25.5 for men and from 23.1 to 24.3 for women during the 24-year study period. The annual change by age group was highest in the ages of 32–39, 40–47 and 48–55 years among men, and in the ages of 24–31, 32–39, and 40–47 years among women. The highest annual changes were found in the youngest birth cohorts for both men and women, i.e., those born 1958–65, 1966–73, and 1974–81. For each birth cohort, the annual change in BMI increased compared to the previous, i.e., older, birth cohort. In addition, age-by-cohort interaction tests revealed that the increase in BMI by increasing age was higher in the younger birth cohorts (1966–1989) than in the older ones.

**Conclusions:**

Public health policies should target those age groups and birth cohorts with the highest increases in BMI. For example, younger birth cohorts had higher annual increases in BMI than older birth cohorts, which means that younger cohorts increased their BMI more than older ones during the study period.

## Background

According to the World Health Organization (WHO), obesity has reached epidemic proportions globally, with approximately 1.6 billion persons (aged 15 years old and above) being overweight or obese [1]. In high-income countries, 8.4% of deaths and 6.5% of disability-adjusted life-years are attributable to obesity or being overweight [2], which impose a significant burden on societies through increased health care expenditure [3], lost productivity due to absenteeism, psychological problems and poorer quality of life [4]. Nevertheless, obesity and being overweight, as well as related chronic diseases, may be prevented [1]. In order to develop efficient public health policies and interventions, mean body mass index (BMI) scores need to be assessed not only in the total population, but also in different population groups.

Body mass index (BMI) is a useful population-level measure as it represents a simple index based on height and weight [1]. WHO criteria define overweight as a BMI of at least 25 kg/m^2^ and obesity as a BMI of at least 30 kg/m^2^. Most studies use self-reported weight and height to calculate mean BMI at the population level [5-9] and this is the approach that constituted the basis for the present study.

Studies in many countries indicate that there have been alarming increases in mean BMI during the last few decades. For example, mean BMI in the UK increased from 24.3 to 25.9 in men and from 23.9 to 25.7 in women between 1980 and 1993 [10,11]. Similarly, it was reported that mean BMI for U.S. adults (18–45 years) increased at an average rate of 2.3 kg/m^2^ per decade between 1986 and 2004 [5]. Increases in mean BMI have also been found in other Western countries [6,7,12-15].

Although increases in mean BMI have been recorded in many countries, these trends may take different trajectories in different countries [2] and in different population groups. Understanding country-specific trends and potential differences between population groups in the mean changes in BMI is therefore important. Previous research has indicated that BMI is associated with health-related factors such as self-reported health status, physical activity, and smoking [6,8,12,14,16], as well as sociodemographic characteristics [4,5]. Recently, nationally representative data in the U.S. between 1986 and 2004 showed that the increase in mean BMI was higher in women than in men. However, longitudinal national trends over several decades, with reference to population characteristics and health status, have rarely been examined. In addition, most studies that have evaluated changes in mean BMI over time have been based on repeated cross-sectional or single-cohort designs [6,8,12,17], and few national-level examinations of BMI trajectories over longer time periods have been undertaken in different age groups and different birth cohorts [5]. For example, an increase in mean BMI over time may reflect increasing BMI with increasing age, but it could also imply that certain birth cohorts have a higher annual increase in BMI than the general population. More detailed knowledge on which age groups and birth cohorts who have the highest annual increases in BMI over time may guide clinicians in their preventive work as well as public health strategies.

The first aim of this study was to analyze, longitudinally, whether the annual change in mean BMI, assessed at four occasions between 1980/81 and 2004/05 and for each sex separately, varied within age groups and birth cohorts and whether there was any age-by-cohort interaction. The second aim was to analyze whether this potential change in BMI was influenced after adjustment for the following covariates: education, urbanization, smoking habits, physical activity and chronic diseases.

## Methods

### The Swedish annual level of living survey

The Swedish Annual Level of Living Survey (SALLS), which has been conducted annually since 1974 by Statistics Sweden, i.e., the Swedish Government-owned Statistics Bureau, was used as the data source in this study. The SALLS includes a nationally representative, simple random sample of adult, non-institutionalized persons aged 16–84 years, taken from the Total Population Register of Sweden. Since 1979, there have been questions, which are not repeated annually, that provide information that makes it possible to follow changes in selected fields, such as health, self-reported weight and height, as well as lifestyle factors. Professional interviewers from Statistics Sweden conduct the interviews one-on-one, usually at the respondents’ homes [18]. The data are not publicly available and their use and analysis requires the permission of Statistics Sweden.

A key feature of SALLS is the repeated assessments of the same individuals, i.e., the “panel”. In this study, we included all individuals aged 16–71 years who were assessed in 1980/81, 1988/89, 1996/97 and 2004/05, and who completed the SALLS at least once. In each SALLS assessment, new individuals aged 16–23 years for evaluation were included. The non-response rate increased during the studied period from 20% to 25%. However, the decrease in response rates did not vary between the different subgroups.

### Ethical approval

The study was approved by The Regional Ethical Review Board in Stockholm (approval no. 12/2000).

### Outcome variables

The outcome variable was body mass index (BMI), calculated as weight (kg)/height (m)^2^, and analyzed as a continuous variable. Weight and height were self-reported. Subjects with missing values for either weight or height were excluded (1%).

### Explanatory variables

We chose to include the following explanatory variables for which previous studies have suggested an association with BMI: sex [5,19], age [8,16], urbanization [20], educational level [9,16], smoking habits [6,8], physical activity [9,12], and chronic diseases [16]. All of these variables were measured at each occasion and are included in the models as time-varying covariates.

*Assessment period* comprised four categories: 1980/81, 1988/89, 1996/97 and 2004/05, including all individuals who had completed the SALLS at least once.

### Sex

Separate analyses were undertaken for men and women.

*Age* was categorized as follows: 16–23, 24–31, 32–39, 40–47, 48–55, 56–63, and 64–71. This categorization reflected the eight-year intervals between the assessments. In Table 1, age is centered around the mean age of 44 years (Agec).

**Table 1:** β-coefficients (β) with 95% confidence intervals (CIs) for BMI (rate of change), men aged 16–71 years using mixed models with random intercepts and random slopes

		**Unadjusted**		**Adjusted**	
**Variable**	**Category**	**β**	**95% CI**	**β**	**95% CI**
*Rate of change*					
**Age-centered**	Centered at 44 y	0.126	0.120-0.132	0.122	0.116-0.127
Agec*cohortc		0.00077	0.00034-0.0012	0.00076	0.00033-0.0012
**Agec-squared**		−0.0019	−0.0022- -0.0016	−0.0019	−0.0022- - 0.0016
**Cohort-centered**	Centered at 1954	0.056	0.050-0.062	0.055	0.049-0.061
**Education**	Low			0	
	Middle			0.19	0.07 to 0.32
	High			0.13	0.00 to 0.27
**Urbanization**^**1**^	1			0	
	2			0.31	0.15 to 0.47
	3			0.33	0.17 to 0.50
**Physical activity**	Almost none			0	
	Once a week			−0.10	−0.20 to 0.01
	>Once a week			−0.24	−0.33 to −0.15
**Smoking**	Non-smokers			0	
	Former smokers			0.31	0.16 to 0.45
	Current smokers			−0.18	−0.34 to −0.03
**Chronic disease**	No			0	
	Yes			0.09	−0.01 to 0.18

^1^Residence in: (1) the three largest cities in Sweden; (2) medium-sized towns (population >90,000); and (3) small towns (population 27,000–90,000) and rural areas.

*Birth cohort* was based on the year of birth and comprised the following groups: 1982–89, 1974–81, 1966–73, 1958–65, 1950–57, 1942–49, 1934–41, 1926–33, 1918–25, and 1910–17, which also reflected the eight-year intervals between the assessments. In Table 1, birth cohort is centered around the mean birth year of 1954 (Cohortc).

### Urbanization

This variable was categorized into large cities (the three largest cities in Sweden, i.e., Stockholm, Gothenburg and Malmö), medium-sized towns (population >90 000), and small towns (population 27 000–90 000)/rural areas.

*Educational level* was divided into compulsory school or less (≤9 years); practical high school, i.e., vocational school, (10–11 years); and theoretical high school and/or college (≥12 years).

### Smoking habits

Respondents were divided into three groups: (1) non-smokers (also including those who smoke now and then); (2) former smokers (regardless of when they quit); and (3) current smokers.

*Leisure time physical activity*, which is associated with BMI [21], was divided into three categories: (1) no or some physical activity; (2) physical activity once a week; (3) physical activity more than once a week.

*Chronic disease* comprised two components: presence or absence of a chronic disease and severity of the disease on a four-grade scale. The variable was dichotomized as: no disease or at least one disease of at least moderate severity.

### Statistical analysis

STATA software package was used in the statistical analyses [22].

Descriptive statistics were used to present the distribution of the explanatory variables (Table 2), as well as mean BMI for the explanatory variables (Table 3) by sex and year of assessment. We chose to show mean values instead of median values, although BMI was not normally distributed. However, the difference between means and medians was very small.

**Table 2:** The distribution (%) of the different variables by sex and assessment period (longitudinal samples of the Swedish population from 1980/81, 1988/89, 1996/97 and 2004/05, complete with new individuals aged 16–23 years)

**Variable**	**Men**				**Women**			
	**1980/81**	**1988/89**	**1996/97**	**2004/05**	**1980/81**	**1988/89**	**1996/97**	**2004/05**
***n***	2728	2688	2570	2177	2770	2666	2634	2211
**Age group**								
16-23	16.3	16.3	12.8	12.7	16.2	16.0	12.0	13.1
24-31	17.6	15.5	16.5	13.1	15.9	15.8	16.5	13.0
32-39	19.6	16.1	16.5	16.6	17.0	15.8	16.6	16.2
40-47	13.2	18.1	16.1	15.2	12.3	16.8	16.1	16.1
48-55	10.6	12.4	17.9	14.5	12.9	11.1	16.5	15.3
56-63	12.4	9.5	11.3	17.1	13.0	12.2	11.0	15.4
64-71	10.3	12.1	8.9	10.8	12.7	12.3	11.3	10.9
**Education**								
Low	42.8	32.9	25.5	20.8	48.7	34.6	25.5	19.2
Middle	26.4	31.3	30.9	26.8	31.1	36.0	33.6	26.8
High	30.8	35.8	43.6	52.4	20.2	29.4	40.9	54.0
**Urbanization**^**1**^								
1	30.8	31.0	31.2	34.3	30.5	30.0	31.4	33.5
2	31.5	33.8	36.5	36.0	32.6	34.7	37.4	36.1
3	37.7	35.2	32.3	29.7	36.9	35.3	31.2	30.4
**Physical activity**								
Almost none	50.3	46.4	42.7	39.2	55.0	50.9	44.1	38.1
Once a week	17.6	18.3	18.6	15.6	20.0	19.7	20.7	13.9
>Once a week	32.1	35.3	38.7	45.2	25.0	29.4	35.2	48.1
**Smoking**								
Non-smokers	38.6	43.4	47.8	52.4	52.4	51.6	51.1	51.9
Former	27.3	29.8	33.9	34.1	16.5	19.7	24.7	20.4
Current	34.1	26.8	18.3	13.6	31.1	28.7	24.2	18.7
**Chronic disease**								
No	72.5	71.1	70.4	67.0	68.1	66.6	63.6	60.5
Yes	27.5	28.9	29.6	33.0	31.9	33.4	36.4	39.5

^1^Residence in: (1) the three largest cities in Sweden; (2) medium-sized towns (population >90,000); and (3) small towns (population 27,000–90,000) and rural areas.

**Table 3:** **Mean BMI values (kg/m**^**2**^**) for subjects in the different explanatory variable groups, presented separately according to sex and assessment period (longitudinal samples of the Swedish population from 1980/81, 1988/89, 1996/97 and 2004/05)**

**Variable**	**Men**				**Women**			
	**1980/81**	**1988/89**	**1996/97**	**2004/05**	**1980/81**	**1988/89**	**1996/97**	**2004/05**
***n***	2728	2688	2570	2177	2770	2666	2634	2211
**Overall mean**	24.1	24.4	25.0	25.5	23.1	23.2	23.9	24.3
**Age group**								
16-23	22.1^7)^	22.0^8)^	22.6^9)^	22.7^10)^	21.1^7)^	21.1^8)^	21.6^9)^	21.8^10)^
24-31	23.3^6)^	23.7^7)^	24.2^8)^	24.4^9)^	21.4^6)^	22.3^7)^	22.9^8)^	23.1^9)^
32-39	24.1^5)^	24.3^6)^	25.1^7)^	25.7^8)^	22.3^5)^	22.4^6)^	23.4^7)^	24.4^8)^
40-47	24.7^4)^	25.0^5)^	25.6^6)^	26.4^7)^	23.4^4)^	23.5^5)^	23.9^6)^	24.6^7)^
48-55	25.1^3)^	25.3^4)^	25.8^5)^	26.4^6)^	24.3^3)^	24.2^4)^	24.9^5)^	24.7^6)^
56-63	25.5^2)^	25.8^3)^	26.0^4)^	26.3^5)^	25.1^2)^	25.2^3)^	25.6^4)^	25.2^5)^
64-71	25.2^1)^	25.6^2)^	26.2^3)^	26.2^4)^	25.0^1)^	24.9^2)^	25.7^3)^	25.9^4)^
**Education**								
Low	24.4	24.4	25.1	24.9	23.6	23.7	24.4	24.2
Middle	24.1	24.5	25.5	26.7	22.8	23.3	24.3	25.2
High	23.7	24.2	24.6	25.2	22.1	22.5	23.3	23.8
**Urbanization**^**a**^								
1	23.8	24.1	24.8	25.2	22.6	22.8	23.5	23.7
2	24.2	24.3	25.0	25.5	23.1	23.2	23.9	24.4
3	24.3	24.6	25.2	25.9	23.5	23.6	24.4	24.7
**Physical activity**								
Almost none	24.4	24.9	25.6	26.0	23.6	23.7	24.6	24.9
Once a week	24.3	24.0	24.9	25.5	22.4	22.8	23.8	24.1
>Once a week	23.5	23.8	24.4	25.1	22.5	22.7	23.2	23.8
**Smoking**								
Non-smokers	23.7	23.8	24.6	25.1	23.6	23.3	23.8	24.0
Former	24.7	25.1	25.8	26.2	22.9	23.6	24.5	24.9
Current	24.0	24.3	24.8	25.2	22.4	22.7	23.6	23.9
**Chronic disease**								
No	23.9	24.1	24.8	25.2	22.7	22.8	23.5	23.7
Yes	24.6	25.0	25.6	26.1	23.9	24.1	24.7	25.1

^a^Residence in: (1) the three largest cities in Sweden; (2) medium-sized towns (population >90,000); and (3) small towns (population 27,000–90,000) and rural areas.

Birth cohort:

^1)^1910-1917;^2)^1918-25; ^3)^1926-33; ^4)^1934-41; ^5)^1942-49; ^6)^1950-57; ^7)^1958-65; ^8)^1966-73;^9)^1974-81;^10)^1982-89.

A mixed linear model with random intercepts and random slopes including age, age squared, birth cohort, and the age-by-cohort interaction (adjusted for education, urbanization, smoking habits, physical activity and chronic disease) was used to assess changes in BMI over time, with one model for each sex. We included a random slope in the model because it showed a significant improvement compared to a model with only random intercepts. An unstructured variance-covariance matrix was assumed. The effects of time period do not need to be estimated for a longitudinal “panel” (see above) study, as age and time period represent the same entity, defined by different variables. Instead the focus can be on the age-by-cohort interaction. Age was included as a random effect. Due to non-linear age effects, the variable age-squared was included in the model. The results are presented as β-coefficients (in kg/m^2^) with 95% confidence intervals (CIs) and as a rate of change in BMI, shown separately for each sex (Table 1). Adjusted BMI means were further estimated, based on the models above (Tables 4 and 5). Annual changes by age group and birth cohort were estimated by applying a linear regression model including the predicted BMI-values (y) based on the models in Table 1 and time (x) for each of the age groups and birth cohorts.

**Table 4:** **Adjusted**^**1 **^**mean BMI values (kg/m**^**2**^**) and annual change in BMI (ΔBMI per year by age group and birth cohort, test of trend) in individuals 16–71 years, presented according to age, birth cohort and assessment period (longitudinal samples of the Swedish population from 1980/81, 1988/89, 1996/97 and 2004/05) by the adjusted model in Table**1**Men**

**Variable**	**Age group**							
**Birth cohort**	**16-23**	**24-31**	**32-39**	**40-47**	**48-55**	**56-63**	**64-71**	**ΔBMI cohort**
**1910-17**	-	-	-	-	-	-	25.2	-
**1918-25**	-	-	-	-	-	25.4	*25.6*	ns
**1926-33**	-	-	-	-	25.1	*25.9*	**26.0**	0.042*
**1934-41**	-	-	-	24.7	*25.4*	**25.9**	***26.3***	0.067*
**1942-49**	-	-	24.0	*25.1*	**25.9**	***26.3***	-	0.095*
**1950-57**	-	23.2	*24.5*	**25.6**	***26.4***	-	-	0.13*
**1958-65**	22.0	*23.6*	**25.1**	***26.4***	-	-	-	0.17*
**1966-73**	*22.2*	**24.0**	***25.6***	-	-	-	-	0.21*
**1974-81**	**22.6**	***24.4***	-	-	-	-	-	0.21*
**1982-89**	***22.7***	-	-	-	-	-	-	-
**ΔBMI age group**	0.032*	0.049*	0.068*	0.071*	0.056*	0.033*	0.046*	
	1980/81		*1988/89*		**1996/97**		***2004/05***	

^1^ Adjusted for education, urbanization, smoking habits, physical activity and chronic diseases.

* p<0.05.

Figures are presented in format according to years: 1980/81 plain, *1988/89 italic*, **1996/97 bold**, ***2004/05 bold and italic.***

**Table 5:** **Adjusted**^**1 **^**mean BMI values (kg/m**^**2**^**) and annual change in BMI (ΔBMI per year by age group and birth cohort; test of trend) in individuals 16–71 years, presented according to age, birth cohort and assessment period (longitudinal samples of the Swedish population from 1980/81, 1988/89, 1996/97 and 2004/05) by the adjusted model in Table **1 **Women**

**Variable**	**Age group**							
**Birth cohort**	**16-23**	**24-31**	**32-39**	**40-47**	**48-55**	**56-63**	**64-71**	**ΔBMI cohort**
**1910-17**	-	-	-	-	-	-	25.0	-
**1918-25**	-	-	-	-	-	24.8	*25.3*	0.063*
**1926-33**	-	-	-	-	24.1	*25.2*	**25.9**	0.093*
**1934-41**	-	-	-	23.4	*24.5*	**25.3**	***25.9***	0.11*
**1942-49**	-	-	22.5	*23.7*	**24.7**	***25.2***	-	0.11*
**1950-57**	-	21.3	*22.7*	**23.8**	***24.7***	-	-	0.14*
**1958-65**	20.9	*22.3*	**23.5**	***24.7***	-	-	-	0.15*
**1966-73**	*21.2*	**22.8**	***24.3***	-	-	-	-	0.18*
**1974-81**	**21.6**	***23.0***	-	-	-	-	-	0.17*
**1982-89**	***21.8***	-	-	-	-	-	-	-
**ΔBMI age**	0.040*	0.073*	0.077*	0.048*	0.025*	0.015	0.045*	
	1980/81		*1988/89*		**1996/97**		***2004/05***	

^1^Adjusted for education, urbanization, smoking habits, physical activity and chronic diseases.

*p<0.05.

Figures are presented in format according to years: 1980/81 plain, *1988/89 italic*, **1996/97 bold**, ***2004/05 bold and italic.***

## Results

The distribution of the different explanatory variables for longitudinal samples of the Swedish population from 1980/81, 1988/89, 1996/97 and 2004/05 are presented separately according to sex and assessment period in Table 2. New individuals aged 16–23 years were added at the last three assessment periods, i.e., 1988/89, 1996/97 and 2004/05. There were clear trends in both men and women towards increased educational attainment, increased urbanization, reduced rates of smoking and increased levels of leisure time physical activity for each successive assessment.

In Table 3, the mean BMI values for subjects by the different explanatory variable groups are presented separately according to sex and assessment period. The results show that mean BMI increased from 24.1 to 25.5 kg/m^2^ in men, and from 23.1 to 24.3 kg/m^2^ in women between 1980/81 and 2004/05. During this time period, mean BMI was consistently 1.0 to 1.2 kg/m^2^ higher in men than in women. Furthermore, mean BMI increased over time in almost all subgroups. At each assessment, mean BMI tended to increase with higher age. In order to show the change in BMI over time in each birth cohort, the ten different birth cohorts are marked with numbers 1–10 in superscript. Reading the numbers 1–10 diagonally reveals that the increase in BMI continued over time until the individuals in the separate birth cohorts reached the highest age, i.e., 64–71 years. Those living in smaller towns had higher BMI than those living in larger cities and medium-sized towns. For both men and women, former smokers had higher BMI than current smokers and nonsmokers, and those with at least one chronic disease of at least moderate severity had higher mean BMI than those without a disease. The BMI gradient in education (increasing BMI with decreasing educational attainment) disappeared among men during the course of the study period, and was attenuated in women.

Two mixed linear models with random intercepts and random slopes (one unadjusted and one adjusted for all the explanatory variables) were applied to estimate the individual effects of age and birth cohort on four repeated assessments of BMI. Two models for each sex are presented in Tables 1 and 6. The rate of change is shown as annual change in BMI by age and birth cohort. The unadjusted model included the variables age (age-centered at 44 years of age), birth cohort (cohort-centered at the year 1954) and the interaction age by cohort (agec*cohortc). The variable agec-squared was also included in order to take into account a potentially curve-linear relationship between age and BMI. For men, the increase in BMI by age was 0.126 kg/m^2^ (95% CI= 0.120-0.132) per year above 44 years of age, compared with the reference group (44 years of age) reflecting increasing BMI by increasing age. The increase in BMI by birth cohort in men was 0.056 kg/m^2^ (95% CI= 0.050-0.062) per birth year compared with the reference group (those born in 1954) reflecting increasing BMI for those born in later years, i.e., those in the younger birth cohorts (Table 1). There was also a significant age-by-cohort interaction of 0.00077 kg/m^2^ (95% CI=0.00034-0.0012), which implies that the increase in BMI by increasing age was higher in the younger birth cohorts (1966–1989) than in the older birth cohorts. Slightly smaller coefficients in BMI by age and birth cohort were seen in women than in men (Tables 1 and 6). The age-by-cohort interaction was somewhat weaker among the women (Table 6).

**Table 6:** β-coefficients (β) with 95% confidence intervals (CIs) for BMI (rate of change), women aged 16–71 years using mixed models with random intercepts and random slopes

		**Unadjusted**		**Adjusted**	
**Variable**	**Category**	**β**	**95% CI**	**β**	**95% CI**
*Rate of change*					
**Age-centered**	Centered at 44 y	0.134	0.128-0.141	0.132	0.125-0.139
Agec*cohortc		0.0001	−0.0006-0.0004	0.00007	−0.0006-0.0004
**Agec-squared**		−0.0012	−0.0015- -0.0008	−0.0013	−0.0016- -0.0009
**Cohort-centered**	Centered at 1954	0.050	0.044-0.057	0.053	0.046-0.059
**Education**	Low			0	
	Middle			−0.27	−0.17 to 0.11
	High			−0.20	−0.36 to −0.04
**Urbanization**^**1**^	1			0	
	2			0.34	0.16 to 0.52
	3			0.53	0.36 to 0.71
**Physical activity**	Almost none			0	
	Once a week			−0.16	−0.27 to −0.05
	>Once a week			−0.36	−0.53 to −0.19
**Smoking**	Non-smokers			0	
	Former smokers			0.27	0.10 to 0.44
	Current smokers			−0.36	−0.53 to −0.19
**Chronic disease**	No			0	
	Yes			0.21	0.10 to 0.31

^1^Residence in: (1) the three largest cities in Sweden; (2) medium-sized towns (population >90,000); and (3) small towns (population 27,000–90,000) and rural areas.

The coefficients in the adjusted models were similar to the coefficients in the unadjusted models. All the adjustment variables were associated with the outcome variable with exception for education in women.

In Tables 4 and 5, adjusted means for BMI and annual changes in BMI, based on the adjusted models in Table 1, are presented according to assessment period (grey contrasts), age group, and birth cohort. The mean BMI increased significantly between 1980/81 and 2004/05 in all male and female age groups and birth cohorts, with the exception for the men born 1918–25. However, the men born 1918–25 were only assessed twice. The annual changes within each age group and birth cohort were estimated by linear regression models (BMI and time) and are presented as ΔBMI age group and ΔBMI cohort. The annual change by age group (ΔBMI age group) varied between 0.032 and 0.071 kg/m^2^ in men, and between 0.025 and 0.077 kg/m^2^ in women. Among men, the ΔBMI was highest in the age groups 32–39, 40–47 and 48–55 years. For example, Table 4 shows that 40–47 year old men increased their BMI from 24.7 (men born in 1934–41, assessed 1980/81 at 40–47 years of age) to 26.4 (men born in 1958–65, assessed 2004/05 at 40–47 years of age) during the study period. Among women, the ΔBMI was highest in the age groups 24–31, 32–39, and 40–47 years.

The annual change by birth cohort varied significantly between 0.042 and 0.21 kg/m^2^ in men, and between 0.063 and 0.18 kg/m^2^ in women. There were apparent trends for both men and women: for each birth cohort the annual change in BMI increased compared to the previous, i.e., older birth cohort. The highest annual changes were found in the younger birth cohorts for both men and women, i.e., those born 1958–65, 1966–73, and 1974–81.

## Discussion

In this longitudinal study with four assessments over time, we found significant increases in BMI between 1980/81 and 2004/05 in all studied subgroups of men and women, e.g. age, birth cohort, educational level, urbanization, level of physical activity, smoking status and chronic disease. Overall, BMI increased by 1.4 kg/m^2^ in men and by 1.2 kg/m^2^ in women during the course of the study period. All age groups and birth cohorts (except men born 1918–25) showed significant annual increases in BMI in both men and women. The annual change in BMI by age group was highest in the ages of 32–39, 40–47 and 48–55 and in the ages of 24–31, 32–39, and 40–47, among men and women, respectively. This change in BMI was only to a minor extent influenced after adjustment for education, urbanization, smoking, physical activity and chronic diseases. For each birth cohort the annual change in BMI increased compared to the previous birth cohort (men and women). This partly reflects the younger age of the later birth cohorts. The highest annual changes in BMI were found in younger birth cohorts, i.e., those born 1958–65, 1966–73, 1974–81, for both men and women. In addition, younger birth cohorts had higher annual increases in BMI by increasing age than older ones, i.e., there was a significant age-by-cohort interaction.

Most past studies that have focused on BMI trends over time were based on repeated cross-sectional samples [23,24] or single cohort studies [25,26]. In repeated cross-sectional studies of different samples of a population, it is difficult to determine whether observed weight gain reflects true age effects or merely age differences in the characteristics of the repeated samples. In contrast, we used longitudinal data obtained through four assessments of the same individuals, with new individuals aged 16–23 years being evaluated on each assessment. This approach allowed us to study whether BMI differed according to age group and birth cohort and whether there was an age group-by-birth cohort interaction.

Our observation of an increasing trend in BMI over time is consistent with previous research, although the magnitude of the BMI increase differs between countries. A longitudinal study from the U.S. [27], which examined similar age groups but covered a shorter and later period of time (1989–1996), reported much larger annual increases in BMI, varying from 0.17 to 0.21 for men and from 0.26 to 0.29 for women, than in corresponding age groups in the present study, i.e., from 0.04 to 0.13 for men and from 0.10 to 0.14 for women. The change in BMI in a Norwegian longitudinal study was also larger than the one in our study [28]. A Dutch study using mixed models (similarly to our study) but with shorter follow-up, also showed an effect of both age group and cohort on BMI [29].

Although possible explanations behind the “better” picture in Sweden compared to, for example, the U.S., were not examined in the present study, it is possible that certain health care strategies have succeeded in preventing even larger increases in BMI in the Swedish population. For example, medical prescriptions of physical activity for primary health care patients by physicians and other health care workers has received positive reviews from both physicians and patients [30].

The change in BMI over time within birth cohorts might partly be due to the fact that younger persons gain more weight. Nevertheless, the increase in BMI in the Swedish population over time cannot be ignored. In our study, mean BMI in 2004/2005 was found to be 25.5 kg/m^2^ (overweight) in men, and 24.4 kg/m^2^ (close to overweight) in women, which reflects the significance of the weight-related problems in Sweden. It is not possible to predict the clinical significance of these increases in BMI at the individual level. However, at a population-level, these increases may have consequences for public health, since high BMI has been associated with dyslipidemia, insulin resistance, the metabolic syndrome, type 2 diabetes [31], low-grade inflammation [32,33], cancer [34], and accelerated aging [35,36]. The burden of disease attributable to excess BMI among adults in the European Region amounted to more than 1 million deaths and about 12 million life-years of ill health (DALYs) in 2000 [37].

Physical inactivity plays an important role in increased BMI. A previous Swedish study showed that men who became physically inactive had a higher increase in BMI between 1980–81 and 1988–89 than those who were physically active on a regular basis [21]. In addition, four independent, cross-sectional population surveys (the FINRISK Studies) conducted in Finland between 1982 and 1997 showed that the inverse association between level of leisure-time physical activity and BMI was significantly strengthened over the 15-year period in both sexes [38].

### Limitations and strengths

This study has some important limitations. One limitation is that our outcome measures were based on self-reported assessments of height and weight, which may have led to underestimated absolute BMI values [39]. However, objectively measured and self-reported height and weight are highly correlated according to a study by Kuskowska-Wolk et al. [40]. The level of self-report bias is probably the same for all four assessments, thus most likely resulting in correct estimates of change between the periods. Any bias due to the subjective nature of our data is therefore most likely conservative.

Another limitation is that the non-response rate was 20-25%. Missing individuals might to a greater part consist of extremes both among underweight and obese individuals. In addition, the sample size was rather large and therefore the sample was divided into subgroups, where non-response bias may have greater effects. On the other hand, the non-response rate in this study was relatively low compared to many surveys from other countries. Moreover, the decrease in response rates did not vary between the different subgroups. Another limitation of the study is that any loss to follow-up might result in selection bias.

This study also has several strengths. Key strengths are the follow-up of BMI changes in individuals for a long period of time (24 years) together with the repeated measurements of BMI. A second strength is that the SALLS is one of the most comprehensive national surveys to date and has been conducted in Sweden for more than thirty years. The sample size is large and, unlike many surveys, each SALLS represent a simple random sample with a longitudinal ”panel” with repeated measurements, drawn from the Total Population Register, and is thus representative of the entire Swedish population. An advantage of longitudinal studies is the possibility to distinguish changes over time within age groups and within birth cohorts.

The surveys in the present study were mainly conducted in the respondents’ homes as face-to-face interviews by well-trained interviewers. The reliability of the survey questions has been estimated by re-interviewing a sample of the participants (test-retest method). The kappa coefficients were 0.64 for self-rated health, and 0.58 for physical activity [41].

## Conclusions

Community and health care interventions should strive to counteract the increases in BMI in the entire Swedish population, and these should particularly target those age groups with the highest annual increases in BMI as well as younger birth cohorts, who had higher annual increases in BMI than older ones. In addition, the significant age-by-cohort interaction implies that the increase in BMI by increasing age was higher in the younger birth cohorts than in the older birth cohorts, which may have significant consequences for public health.

### What this study adds

The novelty of this study comes from the long follow-up of a representative national sample of the Swedish population. The study used longitudinal data obtained through four assessments of the same individuals over a 24-year period, with new individuals aged 16–23 years on each assessment. This approach allowed the study of whether BMI differed according to age group and birth cohort and the age-by-cohort interaction.

The results are important for public health professionals and clinicians, because they suggest that younger birth cohorts have gained more weight over time, which is of particular concern. Community and health care interventions should strive to counteract the increases in BMI in the entire Swedish population, and should particularly focus on the youngest birth cohorts born during the late 50s and later.

## Competing interests

The authors declare that they have no competing interests.

## Authors’ contributions

JS, KS and SJ initiated the project; OKC, SC, PM, JS, KS, SJ worked on conception/design of the study; SJ wrote the initial statistical analysis plan; OKC, SC, PM, JS, KS contributed to the statistical analysis plan; SJ analysed the data; OKC, SC, PM, JS, KS contributed to the analysis and interpretation of the data; OKC drafted the paper; OKC, SC, PM, JS, KS, SJ worked on further drafting and revising the paper critically. The final version of the manuscript to be published was read and approved by OKC, SC, PM, JS, KS, and SJ. All authors read and approved the final manuscript.

## Pre-publication history

The pre-publication history for this paper can be accessed here:

http://www.biomedcentral.com/1471-2458/13/893/prepub
